# Nanoemulsion Encapsulation of Fat-Soluble Vitamins: Advances in Technology, Bioaccessibility and Applications

**DOI:** 10.3390/foods15010156

**Published:** 2026-01-03

**Authors:** Ting Zeng, Fei Song, Zhen Yang, Xianghui Yan, Lianzhou Jiang, Dongze Li, Zhaoxian Huang

**Affiliations:** 1 Key Laboratory of Food Nutrition and Functional Food of Hainan Province, School of Food Science and Engineering, Hainan University, Haikou 570228, China; zengting@hainanu.edu.cn (T.Z.); zyqd2017@163.com (Z.Y.); jlzname@163.com (L.J.); 2 Hainan International Joint Research Center for High Value Processing of Tropical Protein Resources, Haikou 570228, China; 3 Coconut Research Institute, Chinese Academy of Tropical Agricultural Sciences, Wenchang 571339, China; songfeijj@163.com; 4 State Key Laboratory of Food Science and Resources, Nanchang University, Nanchang 330047, China; yanxianghui@ncu.edu.cn; 5 College of Food Science, Northeast Agricultural University, Harbin 150030, China

**Keywords:** nanoemulsion, fat-soluble vitamin, bioavailability, safety, application

## Abstract

This review emphasizes key findings regarding nanoemulsions utilized as carriers for fat-soluble vitamins (FSVs). The stability of FSV-loaded nanoemulsions is chiefly determined by the emulsifier type and concentration, carrier oil type, oil composition and concentration, and droplet size. Natural emulsifiers such as saponins, lecithin, and proteins, in conjunction with long-chain triglycerides (LCT) such as olive and corn oils, prove beneficial in enhancing FSVs’ bioavailability. Despite the established effectiveness of nanoemulsions in enhancing FSVs’ stability and bioaccessibility, the safety of FSV encapsulation within nanoemulsions remains incompletely understood. Importantly, relying solely on in vitro studies is inadequate to simulate the actual gastrointestinal behavior of nanoemulsion systems. Future investigations should prioritize encapsulating FSVs in natural emulsifier-stabilized nanoemulsions and incorporate both in vivo and in vitro experiments to explore the gastrointestinal destiny of these delivery systems. This review offers crucial insights for the systematic development of FSV-enriched functional foods utilizing nanoemulsion technology.

## 1. Introduction

Vitamins are indispensable micronutrients that are crucial for physical growth and development. They are classified as either fat- or water-soluble, depending on their solubility. Compared to water-soluble vitamins, fat-soluble vitamins (FSVs) like vitamin A, vitamin D, vitamin E and vitamin K have more diverse and complex physiological functions. These functions are important for maintaining visual function (vitamin A) [1], regulating calcium and phosphorus homeostasis (Vitamin D) [2], promoting anti-inflammatory and antioxidant properties (Vitamin E) [3], and promoting blood coagulation (Vitamin K) [4].

However, FSVs contain a large number of unsaturated bonds and active groups, which make them prone to oxidation, decomposition, or isomerization reactions under high temperature, light, oxygen, and alkaline conditions, thereby affecting their physicochemical properties and physiological functions [5]. On the other hand, the low water solubility and low bioavailability of FSVs constrain their application in functional foods and nutraceuticals. To tackle these challenges, microencapsulation and nanocapsulation technologies have been investigated to fabricate delivery carriers for FSVs, thereby enhancing their digestive stability and bioavailability [6]. Among them, nanoemulsions have drawn extensive attention from researchers because of their distinctive physicochemical properties and efficient encapsulation capabilities. Compared with conventional emulsions (with droplet sizes typically at the micrometer level), nanoemulsions are nearly thermodynamically stable. They usually possess high optical clarity, excellent physical stability and better oral bioavailability [7]. Table 1 presents a detailed contrast between the characteristics of conventional emulsions and nanoemulsions. The droplet size of nanoemulsions is believed to provide greater kinetic stability and resistance to emulsion instability phenomena such as sedimentation or coalescence. Additionally, the small droplet volume and large specific surface area not only enhance the dispersion and solubility of lipophilic active components but also expedite their release and absorption in the gastrointestinal tract. This dual mechanism significantly improves the bioavailability of FSVs.

Recently, there has been a strong upsurge in the research of delivering FSVs using nanoemulsions with different sizes and structures [8,9,10,11]. These studies aim to maximize the retention rate and bioavailability of FSVs during in vitro digestion. The composition of the carrier oil has emerged as a pivotal factor influencing bioaccessibility. For example, Zhang et al. [12] emphasized that the nanoemulsion containing corn oil as the oil phase exhibited the highest bioaccessibility, with the fish oil system and medium-chain triglycerides (MCT) system following. This observation primarily stems from the formation of larger micelles and hydrophobic regions by long-chain fatty acids (LCFAs), which possess greater solubility, in contrast to medium-chain fatty acids (MCFAs) that exhibit the opposite effect. Similarly, Yao et al. [13] reported that the structural composition of the oil phase is crucial for β-carotene bioaccessibility. Their results showed higher bioaccessibility with LCT nanoemulsions (25.2%) compared to MCT systems (9.8%). Moreover, the study suggested that pre-homogenization mixing of MCT and LCT oils further boosts carotenoid bioaccessibility, reinforcing the significant impact of carrier oil selection on FSV encapsulation in nanoemulsions. Emulsifier choice presents another critical factor optimizing nanoemulsion efficacy. Yuan et al. [14] investigated the effects of three emulsifiers, sodium caseinate, Tween 20, and octenyl succinic anhydride (OSA) modified starch, on the degradation and bioavailability of β-carotene in spinach puree during in vitro digestion. The authors showed that the nanoemulsion stabilized with casein acid had the highest bioavailability (28.8 ± 2.9%), which was significantly better than Tween 20 (13.8 ± 1.1%) and OSA modified starch system (8.1 ± 0.2%). Its absorption also shows the same trend, SC (12.0%) > Tween 20 (5.0%) ≈ OSA-starch (2.6%). However, the bioavailability of FSVs is correlated with a multitude of factors, including release from the food matrix, dissolution in mixed micelles, and interactions with other food components [15]. Initially, the lipid phase encapsulating FSVs undergoes digestion, subsequently merging with bile salts, phospholipids, and other lipophilic substances to form mixed micelles. These micelles proceed through the gastrointestinal mucous layer, reaching epithelial cells for absorption. Interactions with certain food components can inhibit this process. For instance, chitosan can induce mixed micelle precipitation, reducing the bioavailability of vitamin D by nearly 37% [16]. Similarly, Biehler et al. [17] identified that multivalent cations (such as calcium and magnesium) can form insoluble soaps with LCFAs, reducing the number of mixed micelles and thus decreasing the bioavailability of carotenoids. Despite mounting evidence highlighting various formulation parameters’ effects on pre- and post-ingestive stability, a comprehensive systematic review synthesizing these influences remains absent. Thus, the need for a thorough investigation becomes evident, potentially illuminating pathways to optimize nanoemulsion formulations for enhanced FSVs delivery.

As mentioned above, this paper first summarizes and describes the health benefits of FSVs and the different methods of preparing vitamin nanoemulsions. Subsequently, it focuses on the factors influencing the digestive stability and bioavailability of FSVs in nanoemulsion carriers, along with safety considerations. Lastly, it emphasizes the performance of these formulations in food applications. This knowledge is of critical importance to the development of nanoemulsion-based approaches for designing next-generation functional foods applications enriched with FSVs.

**Table 1:** Comparative Analysis of Properties Between Conventional Emulsions and Nanoemulsions [18,19].

	Conventional Emulsion	Nanoemulsion
Droplet diameter	>500 nm	10–500 nm
Thermodynamic stability	unstable	approaching thermodynamic stability
Kinetic stability	unstable	stability
Appearance	turbid to opaque	transparent or translucent or milky liquid
shape	spherical	spherical
polydispersity	often high (>40%)	typically low (<10–20%)
rheological properties	pseudoplastic/plastic flow	general Newtonian flow
emulsifiers	surfactants	surfactants plus co-surfactants

## 2. Fat-Soluble Vitamins

### 2.1. Vitamin A

**Figure 1:**
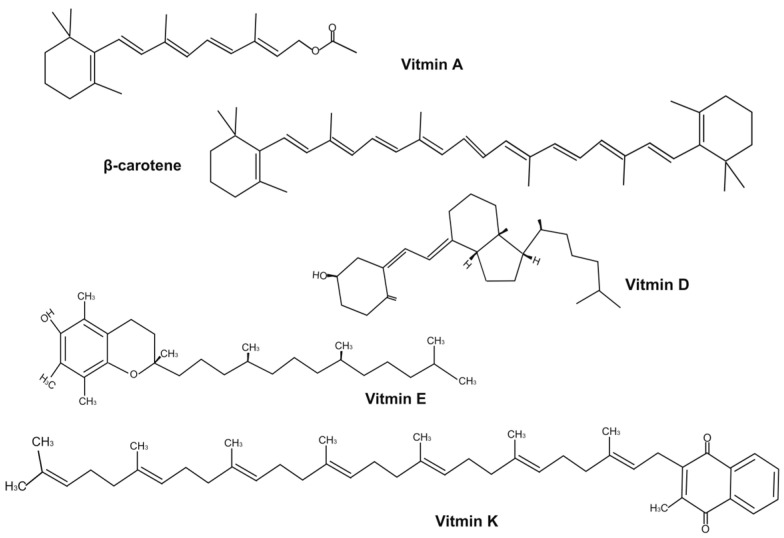
Structural Characteristics of FSVs.

Vitamin A, which includes retinol, retinene, retinoic acid, and retinyl acetate, is a series of retinol derivatives obtained from different food sources. However, Vitamin A is mainly in the form of preformed Vitamin A (retinol) and provitamin A (carotenoid) [20]. As illustrated in (Figure 1), Vitamin A (retinyl acetate) possesses a cyclohexene ring, a conjugated tetraene side chain, and an acetate group, which can be hydrolyzed in vivo to yield active Vitamin A (retinol). Retinol is primarily obtained from a variety of animal-derived foods, including liver, fish oil, eggs, meat, and dairy, and is metabolized into retinene and retinoic acid as required [21]. The structure of retinol plays a pivotal role in vitamin A activity, as it contains an electron-dense region that can scavenge reactive species (e.g., free radicals), thereby attenuating oxidative stress [22]. The high chemical reactivity of vitamin A means that it is susceptible to oxidative degradation and/or isomerization upon exposure to light, heat, transition metals and oxidants [23]. Carotenoids, including β-carotene, β-cryptoxanthin, and α-carotene, are abundantly present in orange and yellow fruits and vegetables like mangoes, sweet potatoes, pumpkins, carrots, papayas, and tomatoes. Among these, β-carotene is the most potent Vitamin A precursor, characterized by a polyene chain of 11 conjugated double bonds and a β-ionone ring at each end. It can be symmetrically cleaved into retinene molecules in vivo [5,24,25]. Vitamin A is essential for multiple vital physiological functions within the human body. This nutrient is vital for preserving optimal visual acuity, promoting growth and development, maintaining epithelial tissue integrity, supporting nervous function and enhancing immune system function [26,27,28]. A lack of Vitamin A has been shown to be a contributing factor to the development of night blindness, anemia, diarrhea, damage to epithelial tissue, nerves, and immune system [29,30].

### 2.2. Vitamin D

Vitamin D, a fat-soluble steroid with anti-inflammatory and antioxidant properties [31], has a four-ring steroid structure (A/B/C/D rings) and a long side chain (Figure 1). The two main forms are D_2_ and D_3_, with D_3_ more biologically active; both convert to the same active form (1,25-dihydroxyvitamin D [1,25(OH)_2_D]) in the human body [32]. Naturally present in seafood, egg yolks, mushrooms, and cod liver oil, Vitamin D is essential for calcium-phosphorus balance and bone health. Moreover, it has significant implications for addressing various non-skeletal diseases, including cancer, autoimmune disorders, and cardiovascular diseases [33]. In addition, vitamin D is extremely sensitive to light, oxygen and heat due to its numerous double bonds [34]. Optically, vitamin D is colourless and transparent, which facilitates its incorporation into a wide range of products. Vitamin D deficiency contributes to rickets and achondroplasia [35].

### 2.3. Vitamin E

Vitamin E consists of eight distinct analogues, including four tocopherols (α, β, γ and δ) and four tocotrienols (α, β, γ and δ). Among these, α-tocopherol (α-TOC) is the predominant form in humans, due to its highest abundance and most potent biological activity [36]. As shown in (Figure 1). Vitamin E consists of a chromanol ring paired with a 16-carbon botanical side chain, featuring phenol hydroxyl antioxidant sites. Research has shown that there is a robust body of evidence associating increased intake of Vitamin E with a range of health benefits, including improvements in chronic heart disease, fatty liver disease, coronary heart disease, atherosclerosis, cancer, Alzheimer’s disease, and chronic eye disease [37,38]. The extensive multifunctional applications of Vitamin E are well-documented, and include its use as an antioxidant, anti-inflammatory agent, cholesterol regulator, skin protectant, immune booster, and hormone modulator [39]. As demonstrated by Ribeiro et al. [40], an absence of Vitamin E has been shown to be a contributing factor to the development of both anaemia and diabetes.

### 2.4. Vitamin K

Vitamin K is classified within the terpenoid group [41], mainly comprising two categories: vitamin K_1_ and vitamin K_2_ [4]. Among them, vitamin K_2_ has been demonstrated to exhibit higher biological activity and a longer half-life [42]. As shown in (Figure 1), Vitamin K is defined by its 2-methyl-1,4-naphthoquinone rings and isoprenoid side chains. It is a vital component of the human body, playing a crucial role in blood coagulation and exerting a significant influence on bone metabolism and cardiovascular health [4]. Vitamin K is susceptible to chemical degradation upon exposure to light and alkaline conditions [15], and thus such conditions should be avoided as much as possible. As asserted by Mallenahalli and Rogers [43], Vitamin K deficiency has been demonstrated to result in the onset of bleeding disorders in newborns.

## 3. Preparation of Nanoemulsions

**Figure 2:**
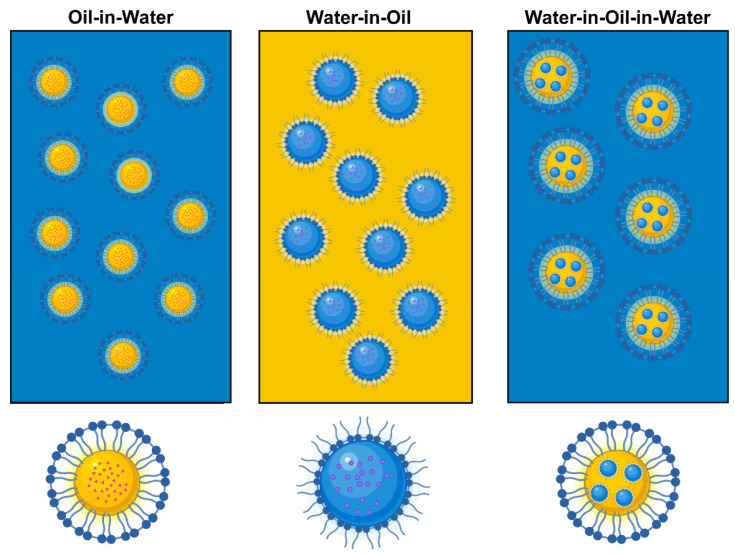
Classification of nanoemulsions. Note: Blue represents the aqueous phase, yellow represents the oil phase, and the small orange and purple particles represent fat-soluble active components.

Nanoemulsion is a colloidal dispersion consisting of one solution dissolved as droplets or bubbles in an immiscible liquid [44]. Despite their kinetic stability, nanoemulsions demonstrate thermodynamic instability and are essentially composed of three essential components: a dispersed phase, a continuous phase, and an emulsifier or surfactant. The proportions and interplay among these components significantly modulate their characteristics and overall physicochemical stability. Nanoemulsions can be categorized into oil-in-water (O/W), water-in-oil (W/O), and multiple nanoemulsions like oil-in-water-in-oil (O/W/O) and water-in-oil-in-water (W/O/W) based on their hydrophilic–lipophilic balance (HLB) values. Typically, HLB values exceeding 10 are employed as a threshold to determine the suitability of emulsifiers/surfactants for classification as O/W nanoemulsions (Figure 2). Emulsifiers/surfactants can generate double-layer and/or solvation forces between dispersed particles, reducing interfacial tension and thus forming stable emulsions [45]. Currently, the upper limit of the average droplet diameter of nanoemulsions is still under discussion. For example, the upper limit of the nanoemulsions is set to 100 nm [9,46], 200 nm [8,47], 500 nm [18,19] or 1000 nm [48] in different review articles. Such differences may stem from the fact that the physicochemical or functional properties of nanoemulsions do not change drastically when droplet size exceeds one of these thresholds. Instead, for different properties, relatively gradual changes are usually exhibited at different particle sizes. For example, nanoemulsions may approach optical transparency in cases where the droplet diameter is less than about 50 nm, whereas nanoemulsions may exhibit extremely high anti-settling stability in cases where the droplet size is less than 500 nm [49]. In this review, we assume nanoemulsions as emulsions whose mean droplet size falls between 10 and 500 nm. This range was chosen because it covers the droplet sizes reported for most nanoemulsion-based FSVs delivery systems in recent literature [50,51], and the corresponding physicochemical attributes (stability, solubilization capacity) effectively meet the requirements for encapsulating and delivering FSVs. Moreover, this size domain constitutes the central focus of the subsequent sections [52].

**Figure 3:**
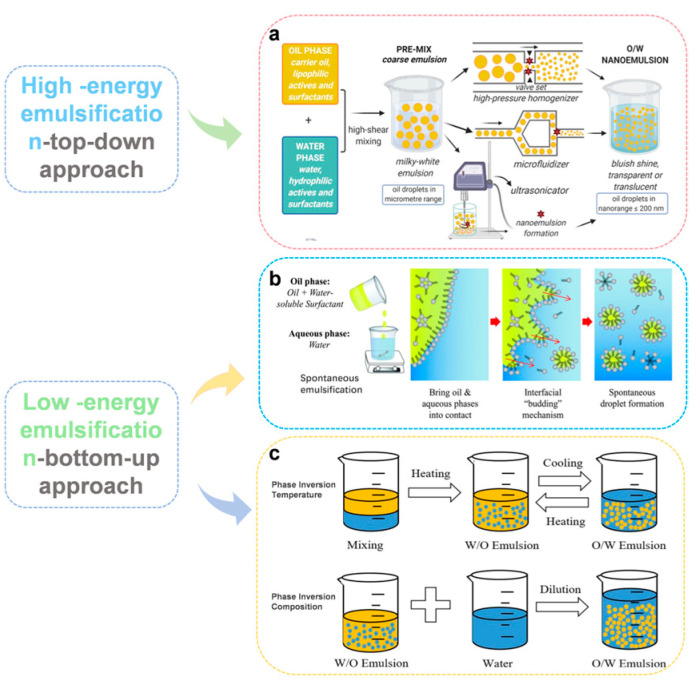
Preparation methods of nanoemulsions for lipophilic vitamins, reprinted with permission of Elsevier Ltd. (Amsterdam, The Netherlands). (**a**) represents high-energy methods, including HPH, USH and MSH, adapted from [54], (**b**) represents SE, adapted from [55], (**c**) represents PIT and PIC, adapted from [46].

The formulation methods of nanoemulsions are usually classified into high-energy emulsification and low-energy emulsification (Figure 3). High-energy methods, including high-pressure homogenization (HPH), ultrasonic homogenization (USH), and microfluidic homogenization (MFH), require specialized equipment. In contrast, Low-energy methods like spontaneous emulsification (SE) and phase transition techniques (Phase inversion temperature PIT and Phase inversion composition PIC) offer energy-efficient ways to create nanoemulsions by leveraging favorable compositional and environmental conditions. However, their requirement for high surfactant-to-oil ratios poses challenges for large-scale industrial adoption due to cost and potential impact on product quality [53]. Currently, high-energy methods are preferred for industrial nanoemulsion production due to their established processes and efficiency in handling large volumes of liquid. These techniques are particularly advantageous for scaling up traditional emulsion practices in food manufacturing.

### 3.1. High-Energy Methods

#### 3.1.1. High-Pressure Homogenization (HPH)

HPH is a highly efficient emulsification technique capable of generating high-intensity shear and turbulence by increasing the pressure and the number of cycles, thereby inducing the emulsified droplets to break up and form smaller particles. Initially, a coarse emulsion, created using a high shear mixer, is passed through a homogenizer where narrow valves apply intense shearing forces to produce a nanoemulsion. The effectiveness of this process is primarily governed by inertial forces in turbulent flows, with laminar shear and cavitation effects also being of considerable importance [56]. The key process parameters affecting HPH emulsification include pressure, number of cycles, and temperature. Generally, as the number of cycles rises, the droplet size correspondingly diminishes [54]. Table 2 sums up recent studies on vitamin-loaded nanoemulsions produced via HPH. For example, Schoener et al. [57] used HPH to encapsulate vitamin D_3_ in three different oil phases, including corn oil, flaxseed oil, and fish oil. They revealed the correlation between the type of carrier oil and the digestion behavior and bioaccessibility of vitamin D_3_. Among the various oils tested, the corn oil-vitamin D_3_ nanoemulsion exhibited the most rapid digestion rate and the highest bioaccessibility of vitamin D_3_. On this basis, Walia et al. [11] further successfully prepared Vitamin D_3_-fortified nanoemulsions stabilized by pea protein by using HPH. These findings revealed a significant reduction in particle size for both 0.5% and 5% *w*/*v* oil samples when the homogenization pressure is elevated from 10 to 20 kpsi. However, further increasing the pressure to 30 kpsi does not yield additional benefits in terms of particle size reduction. Additionally, optimizing homogenization cycles was crucial for minimizing nanoemulsion particle size, as excessive cycling can paradoxically enlarge particles and destabilize the nanoemulsion [32]. Meanwhile, elevated temperatures should be avoided to prevent particle aggregation. The results ultimately indicated that a pressure of 20 kpsi and two cycles are the optimal conditions for the preparation of the stable nanoemulsions. Thus, HPH can be employed to produce vitamin-loaded nanoemulsions, which in turn enhances the characteristics of vitamins. HPH is favored in industrial production due to its ease of operation and scalability, particularly after pre-emulsions. However, the homogenization process is prone to generating high temperatures, which can negatively affect the stability of heat-sensitive components. Moreover, the use of high mechanical power may lead to the disruption of the lipid droplet coatings, thereby affecting the encapsulation efficiency. Therefore, it is necessary to combine low-temperature treatment technologies (such as cooled high-pressure homogenizers) to reduce the loss of heat-sensitive components, and at the same time conduct more research to improve the encapsulation efficiency of active ingredients.

#### 3.1.2. Ultrasonic Homogenization (USH)

USH is a technique that utilizes high-frequency (>20 kHz) sound waves to perturb particles in a sample. By generating destructive forces through high-intensity ultrasound, the oil and water phases are fragmented into extremely small droplets via the cavitation effect. The process of forming nanoemulsions by ultrasonication includes two phases of droplet formation and rupture. The main influencing parameters are ultrasonic power and ultrasonic time. Ultrasonic power directly affects the intensity of the ultrasound, determining the rate at which droplets are broken down in the nanoemulsion system. While ultrasonic time affects the rate of influences the extent of surfactant coverage on the droplet surface, this subsequently impacts the formation of stabilized nanoemulsions. Typically, increased ultrasonic intensity or duration leads to reduced droplet size. Several studies have delved into the nanoencapsulation of FSVs by means of USH (Table 2). For example, Mehmood et al. [68] optimized the preparation conditions of β-carotene nanoemulsions (surfactant concentration, homogenization time, and oil content) using USH. They found that the optimal process conditions for preparing β-carotene nanoemulsions were surfactant concentration of 5.82%, ultrasonic homogenization time of 4 min, and oil content of 6.50%, at which time droplet size 119.33 nm, p-anisidine 2.67, and β-carotene retention 85.63%. Similarly, Jan et al. [60] prepared vitamin D_3_-rich nanoemulsions by using low-temperature-assisted sonication, and optimized the formulation (oil phase, surfactant, co-surfactant) and process parameters (sonication time, stirring rate, and mixing time) of the nanoemulsions, to prepare the optimal nanoemulsions with an average particle size of 169 nm. These studies demonstrate that the USH method can be effectively utilized to prepare vitamin-loaded nanoemulsions. USH is lauded for its simplicity, low energy consumption, minimal external interference, short processing time, and easy maintenance [69]. The disadvantage is that it is only suitable for small-scale laboratory preparation, and large-scale application is restricted [70]. Moreover, USH may cause problems such as protein denaturation and lipid oxidation [71]. In the future, industrial-grade ultrasonic equipment can be developed and the power distribution optimized to avoid local overheating [72]. Use natural antioxidants (such as tea polyphenols) to inhibit lipid oxidation and enhance food safety.

#### 3.1.3. Microfluidic Homogenization (MFH)

MFH is a highly efficient homogenization technique that facilitates droplet disruption by propelling the emulsion pre-mixture through a narrow orifice at high pressure conditions, thereby generating minuscule droplets with a narrow particle size distribution [56]. Its working principle involves dividing the emulsion into two streams, with the continuous and dispersed phases passing through separate narrow channels and then colliding at high speed inside a chamber for interaction. The strong shear forces, turbulence, and cavitation effects generated by this collision form a powerful disruptive force that greatly enhanced the droplet fragmentation efficiency. Research has indicated that the production of droplet size by microfluidizers generally exhibits a downward trend as homogenization pressure, number of cycles, and emulsifier concentration rise [8]. Table 2 summarizes numerous studies that have utilized microfluidization techniques to generate stable nanoemulsions loaded with FSVs. Luo et al. [61] found that emulsifier concentration and homogenization pressure significantly influenced the particle size and stability of β-carotene-rich emulsions prepared via dual-channel microfluidization. As emulsifier concentration increased from 0.1 to 0.5 wt.%, particle size decreased due to better stabilization of oil-water interfaces. With the homogenization pressure increased from 9 to 19 kpsi, the particle size of the emulsion decreased from 204 to 143 nm (using Quillaja saponin) and from 170 to 136 nm (using whey protein isolate). This indicated that higher pressures could provide greater disruptive energy to reduce oil droplet size. Ultimately, the preparation of β-carotene nanoemulsions with a particle size below 200 nm was effectively achieved through using 1 wt.% emulsifier and 15 kpsi homogenizing pressure. On this basis, Agudelo-Cuartas et al. [62] prepared O/W nanoemulsions enriched with α-TOC by microfluidization and applied them in whey protein-based films. The study demonstrated that the average particle size of the nanoemulsion was 113.5 nm, the loss of tocopherol was lower than 30%, and no phase separation occurred after one month of storage at 20 °C. These findings indicate that microfluidization not only effectively reduces particle size but also significantly enhances the stability of the nanoemulsion and the encapsulation efficiency of FSVs. Compared with other homogenization devices, microfluidization requires less surfactant, does not necessitate the pre-preparation of coarse emulsions, and generates a more tightly distributed shear force, allowing the formation of extremely small droplets with fewer cycles. However, it necessitates specific equipment and incurs higher maintenance costs.

### 3.2. Low-Energy Methods

#### 3.2.1. Spontaneous Emulsification (SE)

SE is a technology that through mixing the aqueous phase (water and hydrophilic surfactants) with the organic phase (oil, lipophilic surfactants, and water-soluble solvents) at room temperature to spontaneously form nanoemulsions. During the SE process, the organic phase is gradually added to the aqueous phase. The surfactants rapidly diffuse into the aqueous phase, significantly increasing the interfacial area, thereby forming ultrafine nanoemulsion droplets. The mechanisms may involve interfacial turbulence, diffusion and retention, negative interfacial tension, and dispersion. Table 2 lists several studies in relation to the development of vitamin-loaded nanoemulsions via SE. For instance, Saberi et al. [63] prepared Vitamin E acetate-enriched nanoemulsions through solvent evaporation (SE) using varying concentrations of nonionic surfactants from the TPEG10^®^ series, specifically 20, 40, 60, 80, and 85. Their findings indicated that TPEG10^®^80 provided the most effective emulsification, yielding the smallest particle size of the nanoemulsion. In contrast, TPEG10^®^85 resulted in the largest particle size, which initially decreased and subsequently increased as the surfactant-to-emulsion ratio (%SER) rose, with an optimal concentration identified at 10 wt.%. Meanwhile, the particle size of the nanoemulsion was significantly reduced (from 55 to 48 nm) with the increase in temperature (25–90 °C) and stirring speed of the oil/surfactant mixture. Additionally, the ratio of Vitamin E to MCT in the oil phase significantly affected the particle size. In another study, Guttoff et al. [64] demonstrated that high stirring speed (800 rpm), combined with Tween 80 and a surfactant/oil ratio (SOR) of 1 can form Vitamin D nanoemulsions with particle size less than 200 nm. This finding emphasized that stirring speed can promote the generation of smaller oil droplets and play a key role in promoting homogeneous dispersion of surfactants. Furthermore, Walia et al. [65] prepared Vitamin D nanoemulsions via SE using pea protein-Tween 80 complex, revealing the relationship between the SOR and pH with the nanoemulsion particle size. When the SOR was 1, the emulsion particle size was 134.8 ± 2.2 nm, with good stability and low amount of Tween 80. With the rise in pH, the emulsion particle size gradually increased from 207.7 ± 6.5 nm to 243 ± 51.5 nm, which resulted in the deterioration of emulsion stability. This substantial standard deviation observed with the larger particle size highlights the emulsion’s inherent instability at this pH, underscoring the instability trend with rising pH levels. These studies suggest that vitamin-loaded nanoemulsions with stable and small particle size can be effectively prepared by optimizing the system composition, stirring conditions and environmental factors [73]. SE offers advantages like easy operation, low cost, mild conditions, and high biocompatibility [74]. Nevertheless, it demands precise ratios and preparation conditions, requiring significant quantities of synthetic surfactants, and is primarily suitable for small batch production [75].

#### 3.2.2. Phase Inversion Temperature (PIT)

PIT or the hydrophilic–lipophilic balance temperature is the temperature at which a surfactant’s solubility in oil and water becomes nearly equal. This method utilizes temperature-induced changes in the physicochemical properties of nonionic surfactants, namely optimal curvature and solubility, to induce phase transitions in emulsion systems. Nonionic surfactants are temperature-sensitive. For example, poly-oxyethylene nonionic surfactants exhibit hydrophilicity at low temperatures, with a positive spontaneous curvature, and are suitable for preparing oil-in-water emulsions. At high temperatures, they exhibit lipophilicity, with a negative spontaneous curvature, and are suitable for preparing *w*/*o* emulsions. The emulsions can be transformed into bi-continuous-phase emulsion systems when the system temperature is at the PIT [76]. The PIT method for creating *o*/*w* nanoemulsions involves heating a surfactant-oil-water mixture near or just above its phase transition point, then rapidly cooling it while stirring continuously [77]. For instance, Maurya and Aggarwal [66] formed vitamin D_3_-rich *o*/*w* nanoemulsions by heating a mixture of CCTG, Kolliphor^®^HS15, vitamin D_3_, and an aqueous phase to 85 °C, then cooling to 65 °C, repeating this cycle five times across the phase inversion zone (PIZ). Research has indicated that the oil phase and surfactant concentration significantly affect the emulsion particle size. As the oil phase (from 10% to 25%) increases, the particle size increases from 23.95 nm to 50.96 nm, while the surfactant concentration (from 10% to 40%) increases and the particle size decreases from 48.84 nm to 40.74 nm. Moreover, pH and ionic strength also affect the nanoemulsion particle size, zeta potential, and vitamin D_3_ retention rate. The nanoemulsion particle size and zeta potential varied with pH, with the highest vitamin D_3_ retention rate (69.61%) at pH 7. Increased NaCl concentration led to larger particle sizes and negatively impacted zeta potential and retention rate. This insight suggests that the PIT method is not only effective for the preparation of nanoemulsions, but also can further optimize the stability and functionality of the emulsions by adjusting the chemical environment of the system (pH and ionic strength). The PIT method offers low equipment costs, but it requires extensive use of surfactants and is limited to nonionic surfactants with temperature-sensitive HLB values, narrowing its application scope [78].

#### 3.2.3. Phase Inversion Composition (PIC)

The PIC method involves altering the formulation of the nanoemulsion carrier at a constant temperature, gradually adding one component (water or oil) to the mixture of the other components during the process to obtain a nanoemulsion. The PIC method for fabricating *o*/*w* nanoemulsions comprises two distinct stages. First, the organic phase, composed of oil and surfactant, is mixed to form a stable base. Then, water is added to induce nanoemulsion formation. Analogous to the curvature changes in the PIT technique, the hydration of the surfactant increases upon water addition, shifting its spontaneous curvature from negative to positive. This curvature transition drives the system to form the nanoemulsion by inducing opposing changes. As Dasgupta et al. [67] reported, the PIC method uses edible mustard oil and surfactant Tween-80 as raw materials to effectively encapsulate Vitamin E in the *o*/*w* nanoemulsions. By adjusting the mass ratio of edible mustard oil and surfactant Tween-80 (5:3, 1:1, 3:5, 1:3, 1:7, *w*/*w*), they assessed the impact on the formation and stability of nanoemulsion. The optimal formulation, with 3 *w*/*w*% mustard oil and 5 *w*/*w*% Tween-80, resulted in a uniform and stable nanoemulsion with no flocculation after 15 days of storage. Encapsulation efficiency reached 99.65%, and the Vitamin E concentration measured 17.57 ± 1.54 mg/mL. The encapsulation efficiency of Vitamin E was as high as 99.65%, and the actual concentration was 17.57 ± 1.54 mg/mL. These results demonstrate that with careful formulation optimization, the PIC method can produce Vitamin E nanoemulsions with high encapsulation efficiency and stability. The PIC method offers a notable advantage in that it operates without the need to alter the temperature of system, making it possible to achieve large-scale production due to its operational simplicity. However, it requires a higher amount of surfactants compared to some other methods, raising safety concerns for the resulting nanoemulsions. Thus, it necessitates thorough safety evaluations through specialized experiments.

## 4. Impact of Nanoemulsion Encapsulation on FSVs

Regarding the delivery of bioactive compounds, nanoemulsions tend to outperform conventional emulsions in terms of stability, lipid digestibility and bioavailability. These benefits are crucial for developing more effective carriers for bioactive delivery, enhancing the functional performance of food systems [79]. The stability, lipid digestion rate and bioaccessibility of FSVs in nanoemulsions are impacted by multiple process parameters, including homogenization methods, emulsifier properties, type of carrier oil, oil composition and concentration, and droplet size. This section explores the underlying mechanisms by which these factors affect the stability, lipid digestion rate, and bioaccessibility of fat-soluble vitamins (FSVs) in nanoemulsions (Table 3). By understanding these processes, researchers can better design nanoemulsion systems tailored to maximize the delivery and efficacy of bioactive compounds.

### 4.1. Factors Affecting the Stability of FSVs Encapsulated in Nanoemulsions

Parametric conditions such as homogenization temperature, pressure, cycle times, emulsifier type and concentration have a key impact on the physical and chemical properties and stability of nanoemulsion [44,96]. Mao et al. [80] compared MSH with HPH in the formation of β-carotene nanoemulsions, finding that MSH was more efficient. Specifically, MSH produced smaller droplet sizes (123 nm) and enhanced stability compared to the larger droplet size (154 nm) achieved with HPH. This suggests that microfluidiztion may be a superior technique for manufacturing stable nanoemulsions with fine droplets, which is critical for improving the delivery and retention of bioactive compounds. Additionally, their study revealed that increasing the homogenization temperature, pressure, and number of cycles markedly reduces particle size in β-carotene-loaded nanoemulsions. Moreover, the choice of emulsifier is crucial in influencing both the physical and chemical stability of nanoemulsions incorporating β-carotene [97]. This study also indicated that nanoemulsions stabilized with small molecule emulsifiers, such as Tween (TW) and decanoyl monoacylglycerol (DML), exhibited smaller droplet sizes compared to those stabilized with large molecule emulsifiers like octenyl succinate starch (OSA) and whey protein isolate (WPI). After 12 days of storage at 55 °C, the retention of β-carotene in these nanoemulsions varied significantly. Specifically, DML-stabilized nanoemulsions had the lowest retention rate at 15%, whereas WPI-stabilized ones had the highest at 66%. TW and OSA stabilizers showed retention rates of about 53%. This indicates that OSA and WPI probably form a dense interfacial layer on droplet surfaces, effectively resisting mechanical stress. This layer generates electrostatic or steric repulsion to counteract the attractive forces between droplets, thereby markedly enhancing nanoemulsion stability and protecting β-carotene. Similarly, Li et al. [81] reported that after 14 days of storage, β-carotene retention in WPI- and Tween80-stabilized nanoemulsions was 13.3 ± 0.8% and 7.1 ± 0.6%, respectively, while tea polysaccharide conjugate (TPC) nanoemulsions (TPC-14 and TPC-17) had significantly higher retention (65.2 ± 1.4% and 64.5 ± 1.6%). The high stability of nanoemulsions stabilized by TPC may stem from the strong antioxidant activities of TPC-14 and TPC-17. Furthermore, varying emulsifier concentrations notably impacts droplet size and stability in nanoemulsions containing β-carotene and vitamin K_1_. Increasing emulsifier concentrations fortifies the interfacial film, inhibiting droplet aggregation and coalescence, thereby reducing average droplet size and enhancing stability [68,95]. On this basis, Gasa-Falcon et al. [82] systematically compared the influence of four emulsifiers (Tween 20, lecithin, sodium caseinate, and sucrose palmitate) on β-carotene loaded nanoemulsion stability during in vitro gastrointestinal digestion at varying concentrations (2–8%). The results indicated that the particle size of nanoemulsions stabilized with Tween 20 decreased from 0.35 to 0.30 μm, with lecithin showing a reduction from 0.36 to 0.25 μm, and sodium caseinate decreasing from 0.62 to 0.47 μm as the emulsifier concentration increased. In contrast, the particle size of nanoemulsions stabilized with sucrose palmitate increased significantly, rising from 0.29 to 4.73 μm. The study once again emphasizes that the physicochemical behavior of nanoemulsions in simulating gastrointestinal digestion in vitro is highly dependent on the characteristics of their surface emulsifiers. Overall, these studies highlight the essential roles that homogenization techniques, emulsifier selection, and concentration play in optimizing the stability and bioactive compound retention in nanoemulsion systems [46].

### 4.2. Effect on Bioavailability of FSVs Encapsulated in Nanoemulsions

Bioavailability is generally recognized as the proportion of a bioactive compound that enters systemic circulation, a critical factor for maximizing the nutritional impact of FSVs. Nanoemulsions have emerged as valuable tools in boosting the bioavailability of FSVs, utilizing in vitro digestion methods and Caco-2 cell cultures to shed light on these processes. Furthermore, in vivo studies are conducted to elucidate complex gastrointestinal dynamics. In a nanoemulsion delivery system, FSVs are encapsulated within triglyceride-based nanodroplets. As these droplets traverse the digestive tract, they undergo phase transitions influenced by pH, enzymatic activity, and mechanical shear. In the small intestine, triglycerides are hydrolyzed by lipase into free fatty acids (FFA) and monoacylglycerol (MAG), which co-assemble into mixed micelles alongside bile salts and phospholipids, enabling FSVs to migrate to the micelle’s hydrophobic core. These micelles penetrate the mucus barrier, where absorption occurs via endocytosis/passive diffusion into epithelial cells. Intracellular re-esterification of FFAs and MAGs forms triglycerides, which, together with FSVs, are packaged into chylomicrons (CMs). By entering the lymphatic system and bypassing the hepatic first-pass effect, CMs efficiently deliver FSVs into systemic circulation.

Previous studies have revealed that nanoemulsion-based carries can significantly enhance the bioavailability of FSVs in gastrointestinal tract (GIT) compared to free FSVs. For instance, Teixé-Roig et al. [10] indicated that nanoemulsions stabilized by WPI and soybean lecithin (SBL) exhibited higher plasma levels of retinol compared to a control suspension after oral administration, with WPI nanoemulsions outperforming SBL nanoemulsions in terms of retinol bioavailability. In another study, Walia and Chen [11] found that nanoemulsions with a small droplet size exhibited higher transport efficiency of Vitamin D in Caco-2 cells than free Vitamin D suspensions. Similarly, Parthasarathi et al. [92] demonstrated that smaller sized nanoemulsions exhibited less flocculation and agglomeration during digestion compared to larger sized conventional emulsions. In oral rat experiments, the nanoemulsion showed a higher maximum plasma concentration (C_max_) of Vitamin E (11.253 μg/mL), a shorter time to reach the maximum concentration (T_max_) of 2 h, and an area under the curve (AUC0-inf) that was three times higher than that of the conventional emulsion (22.294 μg·mL^−1^-h^−1^). In addition, the nanoemulsions showed good physical stability for both short- and long-term storage at different temperatures. Collectively, these studies provide compelling evidence that nanoemulsions can notably boost the oral bioavailability of FSVs.

Recent studies have highlighted those optimizing elements such as the type of carrier oil, oil composition, emulsifier choice, and droplet size can significantly enhance the bioavailability of FSVs [10,85,91,93]. Therefore, to maximize the nutritional impact of FSVs in food systems, it is of great importance to consider the multifactorial nature of their overall bioavailability.

#### 4.2.1. Carrier Oil Type

Carrier oil type significantly influences the bioavailability of lipophilic bioactive compounds. This is primarily due to the distinct physicochemical properties of free fatty acids produced during the digestion of various carrier oils [98]. LCT in digestible oils are more prone to form mixed micelles than MCT. Mixed micelles formed from long-chain fatty acids have a larger hydrophobic core, which endows them with superior solubilization capacity. By contrast, indigestible oils (e.g., flavor oils including orange oil and lemon oil, essential oils, and mineral oil) cannot be degraded by lipase under simulated gastrointestinal conditions, because they do not contain hydrolyzable triacylglycerols or fatty acids and are composed of structurally diverse organic compounds (monoterpenes, sesquiterpenes, and oxygenates) [99,100]. In addition, some indigestible oils with high water solubility are prone to induce oil separation in nanoemulsions, which further exacerbates their resistance to digestion [101]. These factors not only inhibit the formation of mixed micelles, but also cause most lipophilic FSVs to remain trapped inside the oil droplets, thus significantly reducing the bioaccessibility of FSVs. Monounsaturated triglycerides (STG) exhibit higher free fatty acid (FFA) release rates, degree of lipolysis, and long-chain fatty acid/medium-chain fatty acid (LCFA/MCFA) ratios. During digestion, STG releases more LCFA, enhancing the solubility of FSVs in mixed micelles and stimulating the formation of chylomicrons (CM) by intestinal epithelial cells. This process effectively increases the bioavailability of FSVs as they are transported across cells in the form of lipoproteins (CM and very-low-density lipoprotein). Empirical studies provide further insights. For instance, Qian et al. [83] demonstrated how the type of carrier oil influences lipid digestion and bioaccessibility of FSVs. They found similar digestion rates for LCT and MCT, both superior to those of orange oil. Specifically, LCT nanoemulsions showed a β-carotene bioaccessibility of 68%, MCT showed 2%, and orange oil nearly 0%. Similarly, Studies utilized simulated gastrointestinal models to demonstrate that LCT emulsions were significantly superior to MCT emulsions in total bioaccessibility of Vitamin E and conversion rate of α-tocopheryl acetate to α-TOC [51,93]. Further, Tan et al. [89] analyzed the bioavailability of vitamin D3 in nanoemulsions incorporating different ratios of digestible (soybean oil) and non-digestible (mineral oil) oils. They concluded that nanoemulsions with solely digestible oils achieved the highest bioaccessibility, whereas those with only non-digestible oils had the lowest, with mixed formulations falling in between. In a related study, Guo et al. [90] identified that STG-loaded Vitamin D nanoemulsions were significantly more bioaccessible compared to MCT/LCT formulations, likely due to enhanced LCFA release, which aided Vitamin D solubilization and absorption in intestinal cells. Lastly, Xia et al. [84] found olive oil superior to corn oil in efficiently enhancing β-carotene bioaccessibility, attributed to the high production of monounsaturated fatty acids, which boosted coelic particle formation and secretion, as well as very low density lipoprotein (VLDL) levels.

#### 4.2.2. Oil Phase Composition and Concentration

The composition and concentration of the oil phase significantly affect the bioaccessibility and bioavailability of fat-soluble vitamins (FSVs). Lipids play a crucial role in facilitating the release of bile salts and the synthesis of micelles, which in turn enhance the solubilization of FSVs within these micelles. Salvia-Trujillo et al. [50] investigated how variations in oil composition and concentration influenced the bioaccessibility of β-carotene. At a total carrier oil concentration of 4% *w*/*w*, increasing the long-chain triglyceride (LCT) content in a mixed oil phase (MCT:LCT) from 0% to 100% *w*/*w* initially decreased bioaccessibility but then increased. This pattern was attributed to the presence of undigested oil and alterations in micellar phase solubility. Interestingly, at a reduced total oil concentration of 1% *w*/*w*, the bioaccessibility consistently improved with higher LCT content, likely due to the enhanced solubility of LCFA-rich micelles. Similarly, Tan et al. [86] systems investigated the effects of varying corn oil concentration (2.5–20%, *w*/*w*) on lipid digestion and β-carotene bioavailability. They observed that particle size and the turbidity of mixed micelles increased with rising oil concentrations. From 2.5% to 10% oil concentration, the solubilization capacity of micelles also rose, thereby increasing β-carotene bioavailability from 60.5% to a peak of 93.2%. However, when the oil concentration further increased to 20%, excess long-chain fatty acids precipitated and some β-carotene precipitated, resulting in a decrease in bioavailability to 80.3%. These results indicate that the oil concentration in nanoemulsion affects the bioavailability of β-carotene by changing the digestion, solubilization and precipitation processes. Further supporting these findings, Yao et al. [85] demonstrated that the bioaccessibility and bioavailability of carotenoids are strongly correlated with initial lipid content. An increase in lipid content (0 g < 0.2 g < 0.6 g < 1.0 g) led to corresponding enhancements in bioaccessibility (3.1% < 7.5% < 14.4% < 19.2%) and bioavailability (15.0 < 35.3 < 39.6 < 106.7 ng/mL), likely due to a higher number of mixed micelles available for solubilizing carotenoids, thereby promoting CM formation.

#### 4.2.3. Emulsifier Type

The emulsifier type plays a crucial role in the bioaccessibility of FSVs by changing the surface area of nanoemulsion droplets, optimizing the binding of digestive enzymes to the droplet surface, and altering the stability of the nanoemulsion. A study probed the impact of plant-based (gum arabic GA and saponin QS) and animal-based (WPI) emulsifiers on the production, stability and bioavailability of corn oil-loaded Vitamin E nanoemulsions. They found that nanoemulsions formed using WPI and QS had smaller droplet sizes compared to those produced with GA, with WPI emulsions showing the highest Vitamin E bioavailability. This could be attributed to the high surface activity of QS that inhibited the action of bile acids and lipases, resulting in slower lipid digestion. In addition, GA emulsions showed better stability under extreme pH conditions. Still, their Vitamin E bioaccessibility was lower, possibly because GA interacted with bile acids or free fatty acids, preventing Vitamin E from entering the mixed micelles. The study highlighted that emulsifier type not only affects physical stability but also significantly impacts Vitamin E bioaccessibility [94]. Notably, more recent studies on typical natural emulsifiers have further confirmed their superiority. For instance, nanoemulsions containing FSVs and eugenol (EU) stabilized by octenyl succinic anhydride modified starch (Purity Gum Ultra, PGU)—a natural modified starch emulsifier—exhibited significantly higher retention rates of β-carotene (≈42%) and Vitamin E (≈90%) after 4 weeks of storage at 40 °C compared to those stabilized by synthetic Tween 80 [102]. The enhanced stability of PGU-stabilized nanoemulsions was attributed to PGU’s ability to form a thicker protective layer around oil droplets, which effectively isolates FSVs from external oxidative factors and inhibits their degradation. Sharif et al. [103] demonstrated that β-carotene encapsulated within lipid droplets coated by natural β-lactoglobulin (a globular protein emulsifier) was more stable to chemical degradation than that coated by synthetic non-ionic surfactant (Tween 20). Building on these findings, Teixé-Roig et al. [10] compared the influences of WPI and SBL on the digestive stability and β-carotene bioaccessibility of nanoemulsions. They observed that WPI-based nanoemulsions exhibited smaller average particle sizes and superior digestibility and bioavailability compared to SBL nanoemulsions. These advantages are likely linked to the protective role of antioxidant peptides generated during protein emulsifier hydrolysis by enzymes like pepsin and trypsin, which help safeguard the compounds and enhance bioavailability. These studies highlight that natural emulsifiers outperform synthetic surfactants in protecting FSVs, improving storage stability, and enhancing bioaccessibility. While smaller droplets generally enhance bioavailability, too small a size may increase fat oxidation.

#### 4.2.4. Droplet Size

The correlation between droplet size and digestion kinetics has significant implications for the bioaccessibility of FSVs. Smaller mean droplet diameters are associated with increased rates and extents of lipid digestion. This can be attributed to the larger surface area-to-volume ratio of smaller droplets, which exposes more lipids to pancreatic lipase, thereby accelerating lipid digestion. Salvia-Trujillo et al. [87] found that during the digestion process, smaller lipid droplets degrade faster than larger ones. Additionally, the bioaccessibility of β-carotene showed a pronounced increase corresponding to the reduction in droplet diameter. This is likely because smaller droplets allow more complete digestion of lipids, facilitating the release and absorption of β-carotene. Consistent with these findings, Salvia-Trujillo et al. [91] demonstrated that vitamin D_2_ was most bioaccessible in small droplet emulsions through an in vitro study. These results highlight the importance of optimizing droplet size to enhance the bioaccessibility of FSVs in nanoemulsion formulations.

## 5. Safety

Encapsulation technology is pivotal for functional ingredient delivery, offering advantages in enhancing the stability, bioavailability, and controlled release of FSVs. Among encapsulation methods, nanoemulsion systems have become central to FSVs delivery research due to their nanoscale dispersion characteristics, promising broad applications in functional foods. However, their safety aspects merit careful consideration.

A substantial body of research has highlighted that the increased bioavailability of nanoemulsions loaded with FSVs may lead to adverse health effects in certain populations after excessive intake, especially when the bioactive substances are toxic at high concentrations, which may trigger elevated toxic concentrations in the blood. Kaur et al. [104] found that nanoemulsions based on α-TOC, lemon oil, Tween-80, and tocopherol polyethylene glycol succinate (TPGS) were not associated with any toxic effects in HepG2 cells by sonication with 100% cell viability. However, another study reported a five-fold increase in toxicity of nanoemulsions doped with β-carotene by Caco-2 cytotoxicity assay. This increase was likely due to the oxidation and metabolism of β-carotene to form reactive intermediates of reactive oxygen species (ROS) within the cells, which can interact with various cellular components, leading to DNA damage, lipid peroxidation, enzyme inactivation and/or protein damage or aggregation [105]. While enhanced bioavailability offers benefits, it also presents health risks if overdosed, especially with certain active substances, necessitating meticulous safety assessments.

Another potential source of toxicity stems from the types and quantities of nanoemulsion constituents, with cytotoxicity particularly arising from surfactants present in the continuous phase of nanoemulsions. Surfactants must fully coat oil droplets to protect bioactive molecules from degradation during gastrointestinal digestion. However, their excessive use may compromise system stability, induce degradation of bioactive molecules, and lead to accumulation in secondary target organs. Jimenez-Escobar et al. [106] demonstrated that serum ALT and AST levels in treatment groups administered β-carotene-loaded conventional emulsions (CE) and nanoemulsions (NE) showed no statistically significant difference or were even lower compared to the control group, indicating the safety of both CE and NE at the administered doses. In contrast, the group treated with the synthetic emulsifier Tween 40 exhibited a 32.3% and 54.9% increase in AST and ALT levels, respectively, resulting in liver injury. Owing to the potential toxicity hazards of synthetic emulsifiers, the focus has increasingly shifted towards the application of natural emulsifiers. Compared with traditional synthetic surfactants (such as Tween and Span), natural emulsifiers have significant advantages: (1) better biocompatibility, which can reduce the risk of cytotoxicity; (2) well-defined metabolic pathways, in compliance with food-grade safety standards; (3) outstanding environmentally friendly characteristics. Typical natural emulsifiers include saponins (tea saponin, ginseng saponin), phospholipids (lecithin) and protein-based emulsifiers (whey protein, pea protein). The amphiphilic nature of their molecular structure enables efficient interfacial stabilization, and these natural alternatives are considered safer and environmentally friendly. Therefore, interest in using natural emulsifiers as alternatives to synthetic emulsifiers for the production of food nanoemulsions is increasing. As demonstrated by Lv et al. [94], WPI effectively reduces interfacial tension, stabilizing nanoemulsions over time. Yang et al. [107] evaluated the performance of saponin relative to synthetic surfactant Tween 80, revealing that saponin could form O/W emulsions with sizes less than 200 nm at low surfactant-oil ratios (SOR ≈ 1:10) with good stability. It is concluded that natural surfactants (saponins) are effective surfactants that can replace synthetic surfactants.

It is noteworthy that the safety assessment of FSV-loaded nanoemulsion systems should be established on a comprehensive multidimensional evaluation framework, which should also incorporate regulatory compliance requirements and systematic in vivo toxicity research data. In terms of regulatory norms, nanoemulsion-based food ingredients are subject to stringent supervision in major regions worldwide. The European Union classifies nanoemulsion-containing foods as novel foods, and mandates comprehensive pre-market safety assessments covering toxicological and environmental impact evaluations [108]. The U.S. Food and Drug Administration (FDA) is responsible for the approval and authorization of nano-based food additives. At the same time, nanotechnology-derived agricultural products are jointly regulated by the Environmental Protection Agency (EPA) and the United States Department of Agriculture (USDA), forming a regulatory system with a clear division of responsibilities. In addition, specialized agencies have been set up in Canada, Switzerland, Japan, China, India and other countries to conduct safety reviews of nanomaterials in food, and these institutions collaborate with authoritative organizations including the World Health Organization (WHO) to formulate the corresponding safe usage levels and toxicity testing & analysis protocols [109]. In terms of in vivo toxicity studies, existing research has confirmed that the toxicokinetic properties of engineered nanoparticles (ENPs) exhibit significant particle-type dependence. Even in the absence of acute toxicity, their long-term in vivo safety assessment is still indispensable. Currently, the academic community generally recommends adopting a tiered testing protocol for the assessment: short-term subacute in vivo tests (14/28-day repeated dosing) and long-term acute toxicity tests (90-day exposure) are performed in classic animal models such as rats and mice to systematically detect the histopathological changes, inflammatory responses, bioaccumulation risks, and absorption, distribution, metabolism and excretion (ADME) profiles induced by nanoemulsions [110,111]. For example, Sriramavaratharajan et al. [112] found through acute oral toxicity studies that cinnamon leaf nanoemulsions (EN) only caused transient salivation at a dose of 2000 mg/kg body weight, whereas no toxic effects were observed at 550 mg/kg body weight, and no macroscopic pathological changes were detected during the 14-day observation period, which confirmed that the nanoemulsions possess both antidiabetic activity and in vivo safety. Silva et al. [113] also verified the non-toxic effects of golden fruit nanoemulsions and their extracts in non-tumor cell lines and *Caenorhabditis elegans* models. In addition to the aforementioned regulatory compliance verification and in vivo toxicity studies, future research should focus on reducing the use of synthetic surfactants and developing food-grade natural emulsifier alternatives, which is the core prerequisite for ensuring the safe and regulatory-compliant application of FSV-loaded nanoemulsions in functional foods.

## 6. Applications in the Food Industry

**Figure 4:**
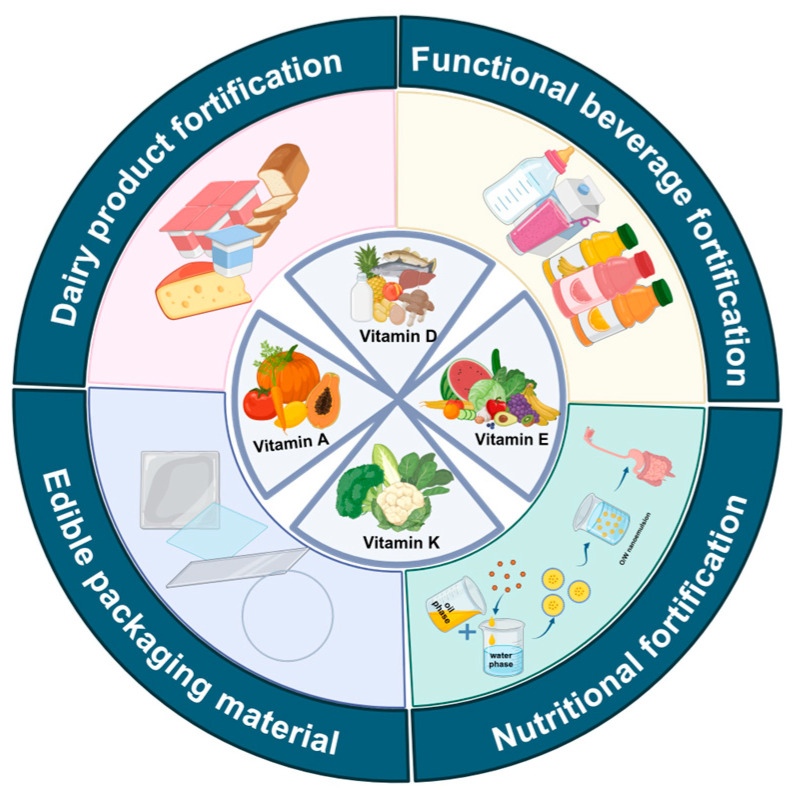
Application of nanoemulsion encapsulation of FSVs.

FSVs have garnered growing interest on account of their diverse array of physiological health activities, such as antioxidant and anti-inflammatory, but their hydrophobicity limits their applications. As an efficient delivery system, nanoemulsions encapsulating FSVs show greater bioactivity than FSVs alone, and owing to their superior stability and ability to enhance bioavailability, they exhibit promising applications in food. The applications of nanoemulsion-encapsulated FSVs are shown below (Figure 4).

### 6.1. Nutritional Fortification

Nanoemulsions can effectively encapsulate FSVs and improve its retention in food. For instance, Mehmood et al. [114] used mixed surfactants (soybean lecithin and Tween 80 are 2:3) as emulsifiers to prepare Vitamin D-loaded nanoemulsion by USH, and optimized the preparation conditions of Vitamin D nanoemulsion (ultrasonic homogenization time, ratio of surfactant to oil, and volume of dispersed phase). Results indicated that a droplet size of 112.36 ± 3.6 nm and a growth rate of 0.141 ± 0.07, with the highest retention rate of vitamin D at 76.65 ± 1.7%, were achieved with 4.35 min of homogenization, a surfactant-to-oil ratio of 0.62, and a dispersed phase volume of 7%. Similarly, Borba et al. [115] developed β-carotene nanoemulsions stabilized with Spectra 80 and Tween 20 using HPH at 10,000 psi for 6 cycles. The average size of the nanoemulsion was about 300 nm, which is within the characteristic range (50–500 nm) of industrial nanoemulsions. Remarkably, after 90 days of storage under various light exposures and temperatures (4, 25, and 37 °C), the emulsions maintained excellent stability and anti-aggregation properties, exhibiting a high retention rate of 70% to 80%. These studies demonstrate that nanoemulsions can significantly enhance the stability and retention of encapsulated FSVs, thereby improving their bioavailability and supporting nutritional fortification in food applications.

### 6.2. Dairy Product Fortification

Nanoemulsion based on FSV encapsulation can be added to dairy products such as buttermilk and mayonnaise to ensure that FSVs are not degraded and enhance bioavailable to consumers. For example, Maurya and Aggarwal [66] explored Vitamin D_3_ fortification in dairy products using a nanoemulsion prepared by the PIT method. Their formulation, consisting of 30% polyethylene glycol hydroxyl stearate (Koliphor^®^HS15), 20% medium-chain triglycerides (CCTG), and 50% water, achieved higher encapsulation efficiency and emulsion stability. When this enriched Vitamin D_3_ nanoemulsion was added to buttermilk, sensory evaluations indicated no significant differences in characteristic attributes between the control and fortified samples. The positive acceptance of the Vitamin D_3_-fortified buttermilk underscores its potential for developing dairy products that meet nutritional fortification objectives. Similarly, Khan et al. [116] demonstrated effective Vitamin D encapsulation in mayonnaise using soy protein isolate (SPI) and WPI. After 36 h of freeze–thaw stability testing, they found that regular mayonnaise exhibited the highest instability (30.50 ± 0.37%), followed closely by mayonnaise with unencapsulated Vitamin D (29.32 ± 0.24%). In contrast, the mayonnaise formula with 5% WPI and 5% SPI-embedded Vitamin D showed the lowest instability percentage (21.28 ± 0.16%), indicating superior stability. This formula also achieved the highest overall acceptability in sensory tests and remained stable for four weeks at 20 °C. Furthermore, in vivo experiments revealed significantly higher serum Vitamin D levels (58.14 ± 6.29 nmol/L) in animals consuming this fortified mayonnaise compared to the control group (37.80 ± 4.98 nmol/L). This evidence highlights the potential of nanoemulsions to enhance the nutritional profile of dairy products while maintaining their sensory qualities.

### 6.3. Functional Beverage Fortification

Nanoemulsion based on FSV encapsulation can be added to milk, yogurt and other drinks to promote the strengthening of FSVs in food, thus improving the nutritional value and stability of functional food. For example, Golfomitsou et al. [117] developed *o*/*w* nanoemulsions loaded with vitamin D_3_ by HPH (1000 bar, 10–15 cycles) using water, soybean oil, and a 4% *w*/*w* emulsifier mixture of Tween 20/lecithin. and incorporated it into full-fat milk for fortification. When added to full-fat milk, these vitamin-enriched nanoemulsions demonstrated high stability in terms of fat content, preventing particle growth and gravity separation over 10 days of storage. This stability is attributed to the protective encapsulation layer, which prevents aggregation and phase separation over time, ensuring uniform distribution of nutrients. Similarly, Zhou et al. [118] used natural surfactant (quinya saponin) to prepare vitamin D_3_ nanoemulsion with an average particle size of 0.14 μm. They investigated the effects of organic (nanocellulose) and inorganic (TiO_2_) nanoparticles on the physical and chemical properties and bioavailability of vitamin D_3_ nanoemulsion fortified almond milk and oat milk. Research has found that both types of fortified milk exhibit low bioavailability of vitamin D_3_ (20%). However, due to the strong repulsive force between the negative surface charges, the nanoemulsion remained stable during 7 days of storage, and was found to be suitable for the strengthening of almond milk. Raikos [119] investigated the effect of heat treatments (63 °C for 30 min, 80 °C and 90 °C for 45 s respectively) on the stability of orange oil beverage emulsions loaded with vitamin E (3.5% *w*/*w*) during refrigerated storage. The results demonstrated that all sports drinks fortified with the nanoemulsions achieved satisfactory vitamin E retention rates (≥85%). Specifically, the beverages subjected to shorter high-temperature treatments exhibited the highest stability after 28 days of storage at 4 °C, which is crucial for meeting the processing and shelf-life requirements of sports drinks. Furthermore, Akkam et al. [120] tested the effect of pea protein based nanoemulsion (PPN) and pea protein (UPP) encapsulated with vitamin D_3_ on strengthening common foods such as milk, infant formula, banana milk, fresh orange juice, orange powder juice, etc. Research has found that PPN prepared with a particle size of 21.8 nm protects over 50% of vitamin D_3_ within 24 h, while the addition of UPP only protects about 15% of vitamin D_3_. Additionally, PPN effectively increased Vitamin D_3_ content in all fortified beverages, prevented UV degradation, and did not alter color, viscosity, chemical composition, or antioxidant activity. These studies collectively underscore the potential of nanoemulsions to improve stability and nutrient delivery in functional beverages without compromising quality attributes.

### 6.4. Edible Packaging Materials

Nanoemulsions can be well incorporated into edible films and coatings for potential food packaging applications, and films/coatings composed of biopolymer matrices form a continuous phase that provides monodispersity and stability for nanoemulsion droplets. The increased viscosity of the continuous phase further reduces droplet aggregation, which is conducive to the uniform dispersion of active ingredients in the film matrix [121]. FSV-loaded nanoemulsions can be effectively used in creating edible packaging materials, providing novel benefits in food preservation and fortification. Bamisaye et al. [7] prepared emulsions and nanoemulsions of vitamin D_3_ at two concentrations of 900 and 1800 μg/g using USH (150 W, 2.5 min/20 kHz, 10 min). These were successfully incorporated into papaya seed gum films, demonstrating their potential for fortification. The study revealed that incorporating emulsions and nanoemulsions increased the thickness, opacity, and hydrophobicity of the films, while reducing water vapor permeability, solubility, and moisture content. Mechanical testing indicated that these incorporated films became more flexible, with a decrease in tensile strength (TS). Furthermore, stability tests showed that vitamin D_3_ was better stabilized within the nanoemulsion than the basic emulsion, especially at lower concentrations. This suggests that papaya seed gum films with vitamin D_3_ nanoemulsions serve as ideal edible packaging materials for ready-to-eat foods, handheld snacks, cheese, and sausages, by enhancing FSV intake and extending the shelf life of these products. Rousta et al. [122] developed edible films incorporated with vitamin D_3_-encapsulated nanoemulsions stabilized by natural *Cordia mucilage* via ultrasonic treatment (200 W, 24 kHz, 50 min). The natural *Cordia mucilage* exhibited strong antimicrobial activity against Gram-positive bacteria, with an antioxidant activity of 67.4% and a flavonoid content of 218.2 μg/g. Nanoemulsions with different mucilage concentrations were characterized, and the optimal encapsulation efficiency (91.5%) was observed in the En1 sample. Notably, the edible films loaded with the encapsulated nanoemulsions exhibited reduced water vapor permeability, decreased solubility, improved thermal stability, and enhanced vitamin D_3_ retention over a 14-day storage period. In addition to nutrient fortification, nanoemulsion-incorporated edible packaging also exhibits excellent functional properties in food preservation. Sun et al. [123] prepared oil-in-water (O/W) lavender nanoemulsions using Tween 80 as a surfactant, and the edible films loaded with the nanoemulsions were applied to the preservation of cherry tomatoes, which exhibited remarkable antioxidant and antimicrobial capacities and effectively prolonged the shelf life of cherry tomatoes. Ran et al. [124] prepared cinnamon essential oil Pickering emulsions stabilized by oxidized cellulose nanofibers, and further incorporated the emulsions together with curcumin into gelatin-chitosan composite films. The prepared functional films possessed low water absorption, strong antioxidant activity and ultraviolet barrier properties, as well as a pH colorimetric function. When applied to pork packaging, these films could effectively delay pork spoilage; meanwhile, the freshness of pork could be visually monitored through the color change in the films from bright brown to saddle brown.

Collectively, the incorporation of nanoemulsions into edible biopolymer films can endow the packaging materials with both nutritional fortification and active preservation functions, which is a promising development direction for green and functional edible food packaging.

## 7. Conclusions

This review elaborates on the stability, bioavailability and key influencing factors of nanoemulsion-encapsulated FSVs, as well as their application potential in the food field. High-energy methods (such as high-pressure homogenization and ultrasonic homogenization) are the preferred industrial solutions due to their mature processing and efficient handling of large-scale systems. They can also achieve high bioavailability of FSVs, providing technical support for the large-scale development of functional foods. Although the safety assessment mechanism of nanoemulsions is not yet perfect and the molecular details of mixed micelle formation and lipid rearrangement in the gastrointestinal tract are still unclear, the attention paid to related functional foods is increasing day by day.

Future research should prioritize enhancing the stability of natural emulsifiers and integrating advanced in vivo tracking technologies to optimize and validate in vitro digestion models, which will provide strong support for the development of the next generation of functional foods rich in fat-soluble vitamins.

## Figures and Tables

**Table 2:** Summary of Methods for Preparing Nanoemulsions Loaded with FSVs.

Methods	Surfactant(s) (*w*/*w*%)	Oil (*w*/*w*%)	Vitamin Concentration (*w*/*w*%)	Emulsification Process	DZ (nm)/PDI	ZP (mV)	References
HSH	Lecithin (2 ^a^) and Q-Naturale (0.5 ^a^)	10 ^a^ (VE:orange oil = 1:1)	0.5 ^a^	High-speed blending followed by high pressure homogenization (12,000 psi, 3 passes)	<200/-	−60	[58]
	Pea Protein (2 ^a^)	10 ^a^ (99 ^a^ flaxseed oil/corn oil/fish oil + 1 ^a^ vitamin D_3_)	0.1 ^a^	High-speed blending followed by high pressure homogenization (12,000 psi, 5 passes)	200~550/-	-	[57]
	Pea Protein (1 ^a^) and Soy Lecithin (1 ^a^)	0.5~5 ^a^ canola oil	0.5 ^a^	High-speed blending followed by high pressure homogenization (20 kpsi, 2 cycles)	<350/<0.3	−25	[11]
USH	Tween 80 and soya lecithin (2.64–9.36%)	olive oil (10%)	5.48–10.52%	Mixing with magnetic stirrer (8000 rpm, 7 min) followed by Sonication (20 kHz, 2.98–8.02 min)	119.33 nm/-	-	[59]
	KolliphorRH-40 (600 μL) + Ethylene Glycol (400 μL)	MCT Oil (100 μL)	-	Mixing with magnetic stirrer (1500 rpm, 10 min) followed by ultrasonication (50 kHz, 30 s)	169/0.288	−22.6	[60]
MFH	Quillaja Saponins (1 ^a^) and Whey Protein Isolate (1 ^a^)	10 ^a^ corn oil	0.1 ^a^	Dual-channel microfluidizer (13 kpsi, 1 pass)	<150/-	−22.6	[61]
	Tween 80 (0.30 ^a^) + Span 60 (0.19 ^a^)	1.29 ^a^ α-TOC	1.29 ^a^	Spontaneous emulsification followed by microfluidization (70 MPa, 3 cycles)	<200/-	-	[62]
SE	TWEENÒ 80 (10 ^a^)	10 ^a^ (8 ^a^ VE + 2 ^a^ MCT oil)	0.8 ^a^	Magnetic stirring (500 rpm, 25 °C)	<200/<0.3	-	[63]
	Tween 80 (10 ^a^)	10 ^a^ MCT oil	2.5 ^a^	Magnetic stirring (500 rpm, 25 °C)	<200/<0.3	-	[64]
	Tween 80 (3 ^b^) + Pea Protein (3 ^b^)	3 ^b^ canola oil	1 ^c^	Magnetic stirring (800 rpm, 25 °C)	207.7/0.31	3.7	[65]
PIT	Kolliphor^®^HS15 (10–40 ^b^) + CCTG (10–25 ^b^)	10–30 ^b^ Leciva S70	0.2 ^b^	Magnetic stirring (five temperature cycles, 85 °C–65 °C–85 °C–65 °C–85 °C–65 °C–85 °C–65 °C–85 °C–65 °C–85 °C)	<100/-	<20	[66]
PIC	Tween 80 (5 ^a^)	3 ^a^ Mustard oil	2 ^a^	Magnetic stirring (400 rpm, 25 °C)	86.45 ± 3.61/0.391 + 0.43	-	[67]

^a^ *w*/*w*%; ^b^ *v*/*v*% and ^c^ mg/mL, these expressions are cited from reference [46]. Abbreviations: CCTG, caprylic-/capric triglyceride; DZ, droplet size; ZP, zeta potential; PDI, polydispersity index.

**Table 3:** Summary of influences on the stability and bioavailability of loaded FSVs nanoemulsions.

Bioactive Compound	Fabrication Method	Surfactant(s) (*w*/*w*%)	Oil (*w*/*w*%)	Vitamin Dispersion Method and Concentration (*w*/*w*%)	Emulsification Process	DZ (nm)/PDI	Result	Reference
β-carotene	MSH and HPH	OSA/Tween 20/WPI/TW, DML (10 ^a^)	Medium chain triglyceride (MCT) oil (10 ^d^)	β-carotene was dissolved in MCT (140 °C, several seconds)/1 ^d^	High speed blender (5000 rpm), HPH (100 MPa, 3 passes)/MSH (100 MPa, 3 passes)	<300 nm/(0.12 < PDI < 0.26)	Small molecule emulsifiers produce smaller droplets in nanoemulsions than large molecule ones, but macromolecular emulsifiers have better stability for β-carotene.	[80]
β-carotene	HPH	TPC (2.0 ^d^)/WPI (1.0 ^d^)/Tween 80 (1.0 ^d^)	Corn oil (8 ^d^)	β-carotene was dissolved in corn oil (sonicating 10 min, 50 °C, 30 min)/0.1 ^a^	High-speed shearer (25,000 rpm, 3 min), HPH (75 MPa, 3 passes)	<140 nm/-	The stability in TPC stabilized nanoemulsions significantly higher than Tween 80 and WPI.	[81]
β-carotene	USH	Tween 80 and soya lecithin (2.64–9.36 ^a^)	Olive oil (10 ^a^)	β-carotene was dissolved in olive oil (−)/5.48–10.52 ^a^	Magnetic stirrer (8000 rpm, 7 min), USH (20 kHz, 2.98–8.02 min)	119.33 nm/-	As the surfactant concentration rises, the rate of β-carotene degradation diminishes.	[68]
β-carotene	MSH	Tween 20/lecithin/sodium caseinate/sucrose palmitate (2–8%)	Corn oil (4 ^a^)	β-carotene was dissolved in corn oil (−)/0.5 ^a^	High-speed shearer (9500 rpm, 2 min), MSH (30,000 psi, 5 times)	-/-	The stability and particle size behavior of β-carotene nanoemulsions during in vitro digestion are significantly influenced by the type and concentration of emulsifiers used.	[82]
β-carotene	MSH	SBL (0.25 ^a^/0.75 ^a^)/WPI (0.25 ^a^/0.75 ^a^)	Corn oil (10 ^a^/30 ^a^)	β-carotene was mixed with corn oil (65 °C, 3000 rpm for 1 min; 17,500 for rpm 2 min, sonication bath for 5 min repeated twice; 9000 rpm for 15 min)/20 ^d^	Homogenizer (11,000 rpm, 2 min), MSH (130 MPa, 5 passes)	<500 nm/-	WPI (C_max_685 ng/mL) enhances retinol bioavailability more than SBL (C_max_394 ng/mL) due to better gut absorption.	[10]
VD_3_	HPH	Pea protein (1 ^b^, 5 ^b^ and 10 ^b^)	Canola oil (0.5, 1, 2.5, 5 ^b^)	Vitamin D_3_ was dissolved in canola oil/(11.7 ^e^)	High-speed mixer (30,000 rpm, 2 min), HPH (10, 20 and 30 kpsi, 1–5 cycles)	170–350 nm/(PDI < 0.3)	Pea protein nanoemulsions (P 230) exhibited approximately 5.3-fold higher transport efficiency across Caco-2 cells (Cancer coli-2) compared to free vitamin D suspension; The cellular uptake efficiency was also about 2.5 times higher than that of pea protein nanoemulsions (P 350)	[11]
β-carotene	MSH	Tween 20 (1.5 ^a^)	Corn oil/MCT/orange oil (4 ^a^)	Crystalline β-carotene was dissolved in oil phase (50 °C, <5 min, 1 h)/0.5 ^a^	High-speed blender (2 min), MSH (9000 psi, 3 times)	140–170 nm/-	The bioaccessibility was much higher for LCT (68%) nanoemulsions than for MCT (2%) nanoemulsions.	[83]
β-carotene	MSH	Tween 20 (0.5 ^a^)	Olive or flaxseed oil (4 ^a^)	β-carotene was dissolved in olive or flaxseed oil (−)/0.04 ^b^	High-shear mixer (3 min), MSH (9000 psi, 3 times.	<200 nm/-	β-carotene bioaccessibility was greater for olive oil (65.2%) than for flaxseed oil (47.8%).	[84]
β-carotene	MSH	Tween 20 (1.5 ^a^)	MCT or LCT (1 ^a^/4 ^a^)	β-carotene was dissolved in MCT or LCT (sonicating 1 min, 50 °C, 5 min)/0.5 ^a^	High-shear mixer (10,000 rpm, 2 min), MSH (9000 psi, 3 times)	<500 nm/-	When the oil concentration was 4% (*w*/*w*), the bioaccessibility of the nanoemulsions first decreased and then increased with the increase in LCT content. When the oil concentration was 1% (*w*/*w*), the bioaccessibility increased from about 14% to 86% with the increase of LCT content.	[50]
β-carotene	HPH	Sodium caseinate (1 ^a^)	MCT:LCT = 1:1 (10 ^a^)	β-carotene was dissolved in oil phase (−)/0.6 ^a^	High-shear blender (10,000 rpm, 2 min), HPH (12,000 psi, five times)	<180 nm/(PDI < 0.2)	The bioavailability increased with increasing lipid content.	[85]
β-carotene	MSH	Tween 20 (2 ^d^)	Corn oil (20 ^d^)	β-carotene was dissolved in corn oil (sonication 40 kHz, 1 min, 50 °C, 5 min)/0.1 ^d^	High-shear blender (10,000 rpm, 2 min), MSH (12,000 psi, 3 passes)	<200 nm/-	The bioavailability of β-carotene shows a trend of first increasing and then decreasing with oil concentration	[86]
β-carotene	MSH	Tween 20 (1.5 ^a^)	Corn oil (4 ^a^)	β-carotene was dissolved in corn oil (sonicating 1 min, <50 °C, 5 min)/0.5 ^a^	High-speed blender (10,000 rpm, 2 min), MSH (4 kpsi/9 kpsi, 5 times)	<400 nm/-	β-carotene bioaccessibility was found to decline progressively with a reduction in droplet size, with values dropping from approximately 59% (small emulsion) to 34% (large emulsion).	[87]
VD_3_	HPH	Quillaja saponin (2 ^a^)	Corn oil (10 ^a^)	VD_3_ was dissolved in corn oil (−)/0.1 ^a^	High-speed blender (2 min), HPH (12,000 psi, 3 cycles)	<400 nm/-	In vitro studies showed that the VD_3_ concentration of nanoemulsion was 3.94 times higher than traditional crude emulsion group. In vivo studies have shown that crude emulsion increases serum 25 (OH) VD levels by 36.04%, while supplementing VD with nanoemulsion increases VD levels by 73.10%	[88]
VD_3_	MSH	Whey protein isolate (1 ^d^)	Corn or mineral oil (10 ^a^)	Vitamin D_3_ was dissolved in either corn oil (digestible oil) or mineral oil (indigestible oil) (−)/0.2 ^d^	High-speed mixer (10,000 rpm, 2 min), MSH (12,000 psi, 5 times)	<170 nm/-	The extent of bioaccessibility was markedly greater in the nanoemulsions samples containing solely digestible oil (75.2%) compared to those with only indigestible oil (20.7%).	[89]
VD	HPH	Tween 20 (1 ^b^)	STG or MCT/LCT (10 ^a^)	Vitamin D was dissolved in STG or MCT/LCT (−)/0.1 ^a^	High-speed ultra-Turrax blender (19.2 bar, 2 min), HPH (600 MPa, 5 cycles)	<200 nm/-	In comparison to MCT/LCT (45.40 ± 2.85%), STG (61.31 ± 2.90%) demonstrated a significantly greater VD bioaccessibility.	[90]
VD_3_	HPH	Pea protein (2 ^a^)	Flaxseed oil, corn oil, or fish oil (10 ^a^)	Vitamin D_3_ was dissolved in oil phase (−)/1 ^a^	High-shear blender (2 min), HPH (12,000 psi, 5 passes)	200~550 nm/-	Vitamin bioaccessibility was notably superior in MUFA-emulsions (78%) compared with PUFA-emulsions (43%).	[57]
VD	USH	Tween 80 (20 ^c^ of buffer)	Corn oil20 ^a^	Vitamin D was dissolved in corn oil (magnetic stirrer 30 min)/0.5 ^c^ of oil	High speed blender (10,000 rpm, 2 min), MSH (6000 psi, 15,000 psi, 3 times)	<600 nm/-	In vitro studies have demonstrated that the bioaccessibility of vitamin D is inversely related to droplet size. In vivo studies have indicated that emulsions with the largest droplet size have higher vitamin D absorption.	[91]
VE	MSH	Saponins (0.1 ^d^)	Sunflower oil (10 ^d^)	vitamin E was dissolved in sunflower oil (−)/2 ^d^	High-speed homogenizer (15,500 rpm, 5 min), MSH (12,000 psi, four cycles)	277 nm/-	The bioavailability of nanoemulsions is three times higher than that of traditional emulsions.	[92]
VE	MSH	Q-Natural^®^ (1 ^d^)	Corn oil (LCT) or MCT (10 ^d^)	Vitamin E was dissolved in either corn oil (LCT) or MCT oil (−)/2.5 ^d^	High-speed mixer (2 min), MSH (9000 psi, 4 cycles)	228–270 nm/-	The bioaccessibility and conversion of a-tocopherol acetate to a-tocopherol was markedly greater in LCT (39% and 29%)-emulsions than in MCT (17% and 17%)-emulsions.	[51]
VE	MSH	Q-Naturale (0.5 ^d^)	Corn oil (LCT) or MCT (10 ^a^)	Vitamin E was dissolved in either corn oil (LCT) or MCT oil (−)/25 ^d^	High-speed mixer (2 min), MSH (9000 psi, 5 cycles)	-	The bioaccessibility of LCT-emulsions (46%) than MCT-emulsions (19%) The conversion of α-tocopherol acetate to α-tocopherol was more pronounced in LCT (90%) than MCT (75%).	[93]
VE	MSH	Gum arabic or quillaja saponin or whey protein isolate (1.5 ^a^)	Corn oil (10 ^a^)	Vitamin E was dissolved in corn oil (−)/2 ^a^	(−)/MSH (12,000 psi, 3 times)	-	The bioaccessibility of WPI-emulsions (85%) was higher for other two emulsions (65%).	[94]
VK1	SE	Tween 80 (5–20%)	α-TOC	VK_1_ was dissolved in α-TOC (−)/5.48–10.52%	Organic phase slowly added into an aqueous phase under magnetic stirring at 700 rpm, stirred for 5 min at 1400 rpm.	<300 nm/(PDI < 0.2)	With the increases in the concentration of surfactant, there is a corresponding decline in droplets size.	[95]

^a^ *w*/*w*%; ^b^ *w*/*v*%; ^c^ mg/g; ^d^ wt.%; ^e^ mg/mL, Abbreviations: DZ, droplet size; PDI, polydispersity index.

## Data Availability

The original contributions presented in the study are included in the article. Further inquiries can be directed to the corresponding authors.
